# Disruption of the *C*. *elegans* Intestinal Brush Border by the Fungal Lectin CCL2 Phenocopies Dietary Lectin Toxicity in Mammals

**DOI:** 10.1371/journal.pone.0129381

**Published:** 2015-06-09

**Authors:** Katrin Stutz, Andres Kaech, Markus Aebi, Markus Künzler, Michael O. Hengartner

**Affiliations:** 1 Institute of Molecular Life Sciences, University of Zurich, Zurich, Switzerland; 2 Center for Microscopy and Image Analysis, University of Zurich, Zurich, Switzerland; 3 Institute of Microbiology, ETH Zurich, Zurich, Switzerland; Ghent University, BELGIUM

## Abstract

Lectins are non-immunoglobulin carbohydrate-binding proteins without enzymatic activity towards the bound carbohydrates. Many lectins of e.g. plants or fungi have been suggested to act as toxins to defend the host against predators and parasites. We have previously shown that the *Coprinopsis cinerea* lectin 2 (CCL2), which binds to α1,3-fucosylated N-glycan cores, is toxic to *Caenorhabditis elegans* and results in developmental delay and premature death. In this study, we investigated the underlying toxicity phenotype at the cellular level by electron and confocal microscopy. We found that CCL2 directly binds to the intestinal apical surface and leads to a highly damaged brush border with loss of microvilli, actin filament depolymerization, and invaginations of the intestinal apical plasma membrane through gaps in the terminal web. We excluded several possible toxicity mechanisms such as internalization and pore-formation, suggesting that CCL2 acts directly on intestinal apical plasma membrane or glycocalyx proteins. A genetic screen for *C*. *elegans* mutants resistant to CCL2 generated over a dozen new alleles in *bre 1*, *ger 1*, and *fut 1*, three genes required for the synthesis of the sugar moiety recognized by CCL2. CCL2-induced intestinal brush border defects in *C*. *elegans* are similar to the damage observed previously in rats after feeding the dietary lectins wheat germ agglutinin or concanavalin A. The evolutionary conserved reaction of the brush border between mammals and nematodes might allow *C*. *elegans* to be exploited as model organism for the study of dietary lectin-induced intestinal pathology in mammals.

## Introduction

Lectins are carbohydrate-binding proteins without enzymatic activity towards the bound carbohydrates and are of non-immunoglobulin origin [1]. Apart from diverse internal biological functions in plants, fungi, and animals [1–3], lectins have also been suggested to act as toxins to defend against predators and parasites, likely explaining the observed toxicity of some lectins against various organisms [4,5]. A particular example of such toxins are the dietary lectins, found for example in grain and legumes, which are toxic to humans [6]. For example, the wheat germ agglutinin (WGA) binds to and damages the brush border (microvilli and glycocalyx at the intestinal apical surface [7]) of the intestinal epithelium in mammals [8,9].

Lectins can exert their toxicity in various ways. Some lectins induce toxicity by carbohydrate binding only [10,11], either acting at the membrane directly [12] or sometimes following endocytosis [13]. Other lectins possess an additional domain with enzymatic activity that interferes with cell function. For example, ricin inactivates ribosomes [14], *Marasmius oreades* agglutinin (MOA) degrades crucial internal proteins by its cysteine protease activity [15], while the *Laetiporus sulphureus* lectin (LSL) is an exotoxin that binds to and induces pores at the cell surface [16]. However, for many lectins, the actual molecular mechanism of toxicity remains unknown, even when the bound sugar moiety (glycotarget) and / or the structure of the lectin are known.

The nematode *Caenorhabditis elegans* has been extensively used as a model system to study infection and toxicity mechanisms. *C*. *elegans* can be infected with diverse pathogens such as bacteria, fungi, microsporidia or viruses [17–19], which can kill it by diverse strategies including colonization, persistent infection or invasion of the intestine, biofilm formation, or through the action of toxins such as the pore-forming crystal proteins of *Bacillus thuringiensis* [20,21]. In *C*. *elegans*, pathogens mainly target the intestine or the cuticle / hypoderm. The resulting intestinal disease phenotypes range from lumenal distention, lumenal colonization by bacteria, damaged apical plasma membrane to cell lysis [17,22].

*C*. *elegans* has also been used to study the mechanisms of toxicity of various molecules, including pharmacological agents, heavy metals, and lectins [23,24]. We previously reported that the fungal lectin *Coprinopsis cinerea* lectin 2 (CCL2) is toxic when fed to *C*. *elegans*. CCL2 binds to glycoproteins carrying an α1,3-fucosylated N-glycan core [10]. Biosynthesis of this glycotarget occurs via conversion of GDP-mannose to GDP-fucose by BRE-1 and GER-1 [10], followed by transfer of this fucose to the proximal and distal N-acetylglucosamine (GlcNAc) residues of the N-glycan core by the α1,3-fucosyltransferases FUT-1 and FUT-6, respectively [25]. Complete loss of fucose by mutations in *bre-1* or *ger-1* prevents CCL2 binding and therefore conveys complete resistance to CCL2 ([10] and see below). Failure to attach the proximal fucose by mutations in *fut-1* leads to partial resistance, whereas failure to attach the distal fucose by mutations in *fut-6* still allows for toxicity by binding of CCL2 to the proximal fucose. Complete resistance is achieved in the *fut-6 fut-1* double mutant. CCL2 only has a single carbohydrate-binding site and no known enzymatic activity [10]. Upon feeding to *C*. *elegans*, CCL2 causes developmental delay and premature death [10]; the underlying cytopathology has however not been investigated yet.

Here we present a detailed characterization of the CCL2 toxicity phenotype in *C*. *elegans*. We found that CCL2 binds to the brush border of the intestinal epithelium, leading to dramatic morphological changes within hours, including loss of microvilli, invaginations of the intestinal apical plasma membrane and gaps in the terminal web. Interestingly, the structural defects induced by CCL2 are very similar to those observed at the mammalian intestinal brush border following exposure to dietary lectins such as WGA or concanavalin A (Con A) [9]. In our model organism, the disorganization of the brush border is likely the underlying cause for the observed intestinal malfunction, developmental delay, and premature death. We could exclude known toxicity mechanisms such as lectin internalization or pore formation in the intestinal apical plasma membrane, suggesting that CCL2 exerts its toxicity in a novel way directly at the cell surface. Given the similarity of the observed phenotypes in worms and mammals, *C*. *elegans* could possibly be a good model to better understand the cellular and molecular intestinal pathology induced by dietary lectins in mammals.

## Results

### Exposure of *C*. *elegans* to the fungal lectin CCL2 leads to delayed development, an enlarged intestinal lumen, and premature death

**Fig 1:**
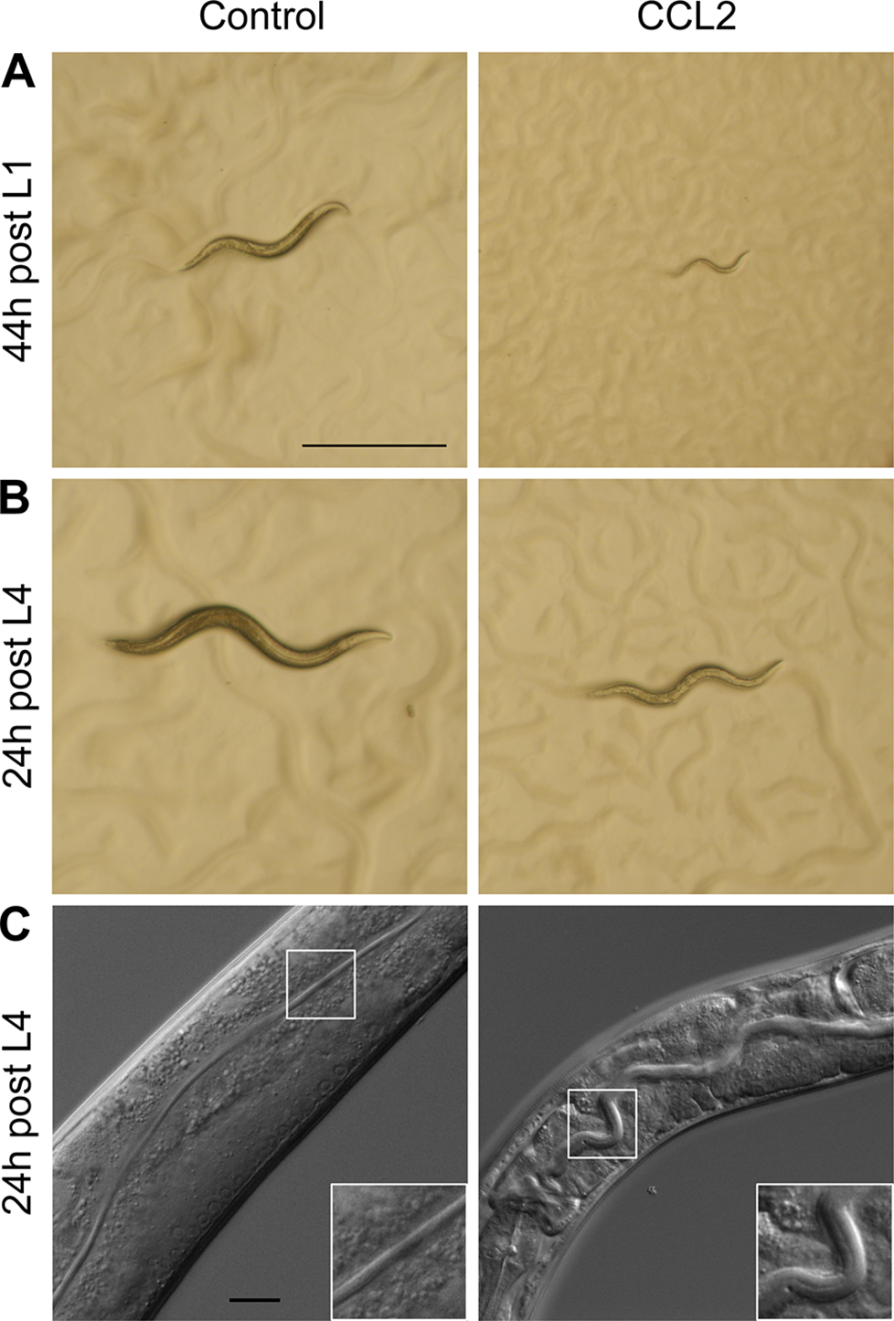
CCL2 exposure to *C*. *elegans* results in delayed development and enlargement of the intestinal lumen. (A) Wild-type *C*. *elegans* fed on control (empty vector) *E*. *coli* reached the L4 larval stage 44 h after hatching, whereas worms fed on CCL2-expressing *E*. *coli* only reached larval stage L2 (stereo microscopy). (B,C) Wild-type L4 larvae kept on control or CCL2-expressing *E*. *coli* for 24 h. The detectable developmental delay was evident already after 24 h (B; stereo microscopy). CCL2 also caused an enlarged intestinal lumen in these larvae (C; DIC microscopy). Scale bar: 0.5 mm (A-B), 20 μm (C); inset: 2x magnification of the lumenal section of the intestine.

To assess the effect of CCL2 exposure on development, wild-type *C*. *elegans* embryos were left to hatch and develop on plates seeded with *Escherichia coli* BL21(DE3) expressing either CCL2 or the empty vector (pet24) as a negative control (Fig 1). Animals raised on control plates reached the L4 stage approximately 44 h post-hatching, whereas CCL2-fed worms only reached the L2 stage during this time (Fig 1A). Worms exposed to CCL2 did eventually reach adulthood on day 7, but looked sick, pale, and produced only few progeny before succumbing approximately 2 days later (3 days post-L4). CCL2 is also toxic to animals exposed at later developmental stages: whereas wild-type L4 larvae fed on control *E*. *coli* for 24 h matured into fertile adults, L4 animals fed on CCL2-expressing *E*. *coli* developed into thin, small, pale, sick young adults that had not yet laid any eggs (Fig 1B). Differential Interference Contrast (DIC) microscopy of CCL2-treated animals revealed a meandering and enlarged intestinal lumen (Fig 1C and S1A Fig). The condition of these animals worsened over time: only few progeny were produced and the animals started dying 3 days post-L4. We conclude that chronic exposure of *C*. *elegans* to CCL2 causes delayed development, a distended intestinal lumen, reduced brood size, and premature death.

### CCL2 binds to and alters the brush border of the intestine without being internalized

**Fig 2:**
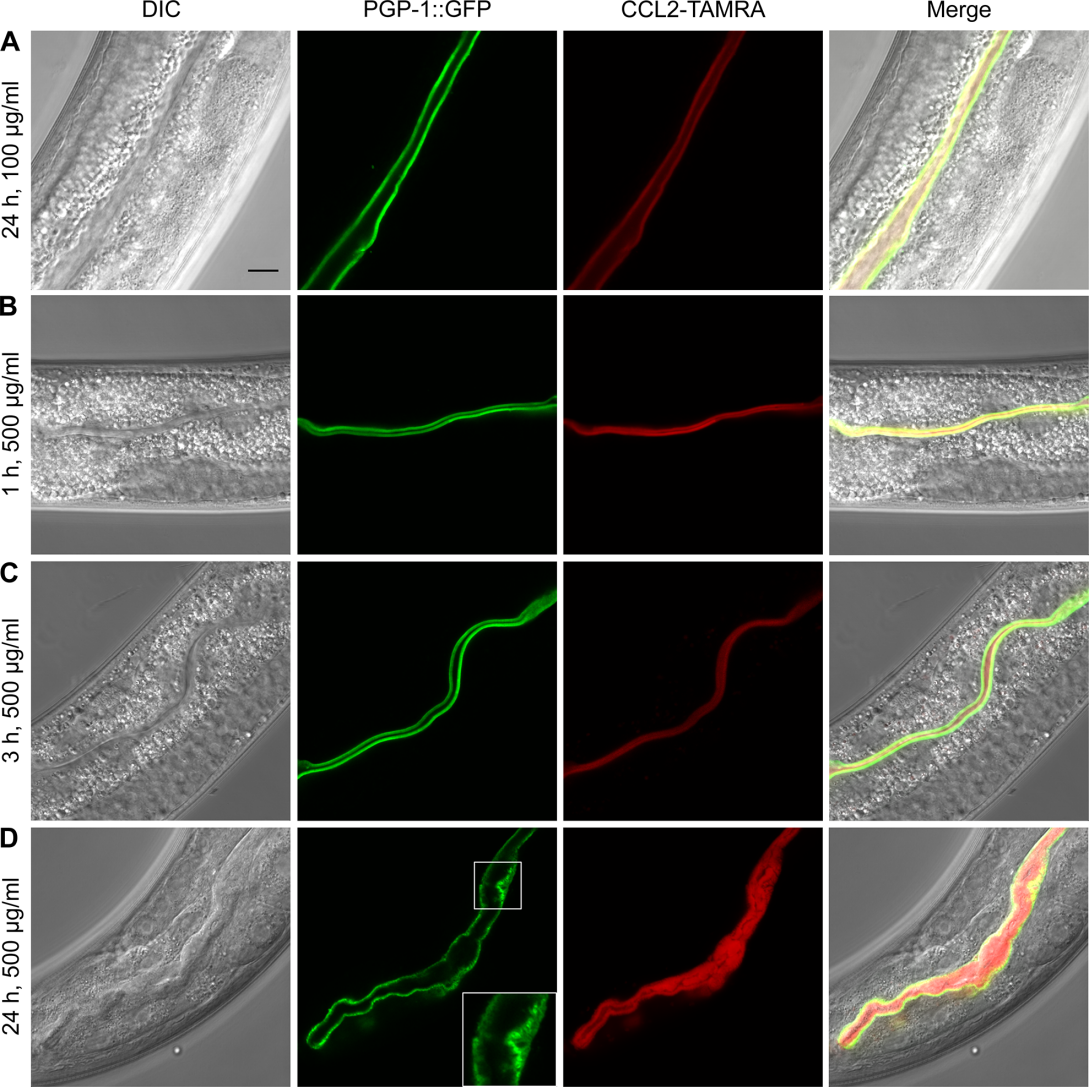
CCL2 binds to and alters the brush border of the intestine without being internalized. (A-D) *C*. *elegans* L4 larvae expressing PGP-1::GFP were fed with a non-toxic (100 μg/ml; A) or toxic (500 μg/ml; B-D) concentration of CCL2-TAMRA (tetramethylrhodamine: red) for the indicated hours and observed using confocal microscopy. (A) CCL2-TAMRA colocalized with PGP-1::GFP at the apical intestinal plasma membrane. (B-D) Toxic concentrations of CCL2-TAMRA led to a time-dependent alteration of the brush border without any detectable uptake into intestinal cells. Scale bar: 10 μm; inset: 2x magnification of the lumenal section of the intestine.

To identify the possible site of action of CCL2, we fed purified red fluorescently labeled recombinant CCL2 protein (CCL2-TAMRA (tetramethylrhodamine)) to *C*. *elegans* L4 larvae expressing PGP-1::GFP [26], an ATP-binding cassette (ABC) transporter that localizes to and marks the intestinal apical plasma membrane (Fig 2 and S1B Fig). CCL2-TAMRA at a non-toxic concentration (100 μg/ml) colocalized with PGP-1::GFP after 24 h, indicating binding of CCL2 at the apical surface (glycocalyx or plasma membrane) of the intestine (Fig 2A and S1B Fig). To determine whether CCL2 was internalized after binding, we also performed a time course experiment using a toxic concentration (500 μg/ml) of CCL2-TAMRA. At all time points, CCL2-TAMRA was confined to the intestinal lumen, showing that CCL2 was not taken up by intestinal cells. After 1 and 3 h, CCL2 bound to the intestinal apical surface without any signs of toxicity (Fig 2B and 2C), whereas after 24 h, the intestinal lumen was distended and the plasma membrane deformed (Fig 2D, inset). Taken together, these observations suggest that binding of CCL2 to the *C*. *elegans* intestinal apical surface is sufficient to cause the developmental delay and the changes observed at the intestinal apical plasma membrane.

### Exposure to CCL2 results in loss of microvilli, deformation of the intestinal apical plasma membrane, and gaps in the terminal web

**Fig 3:**
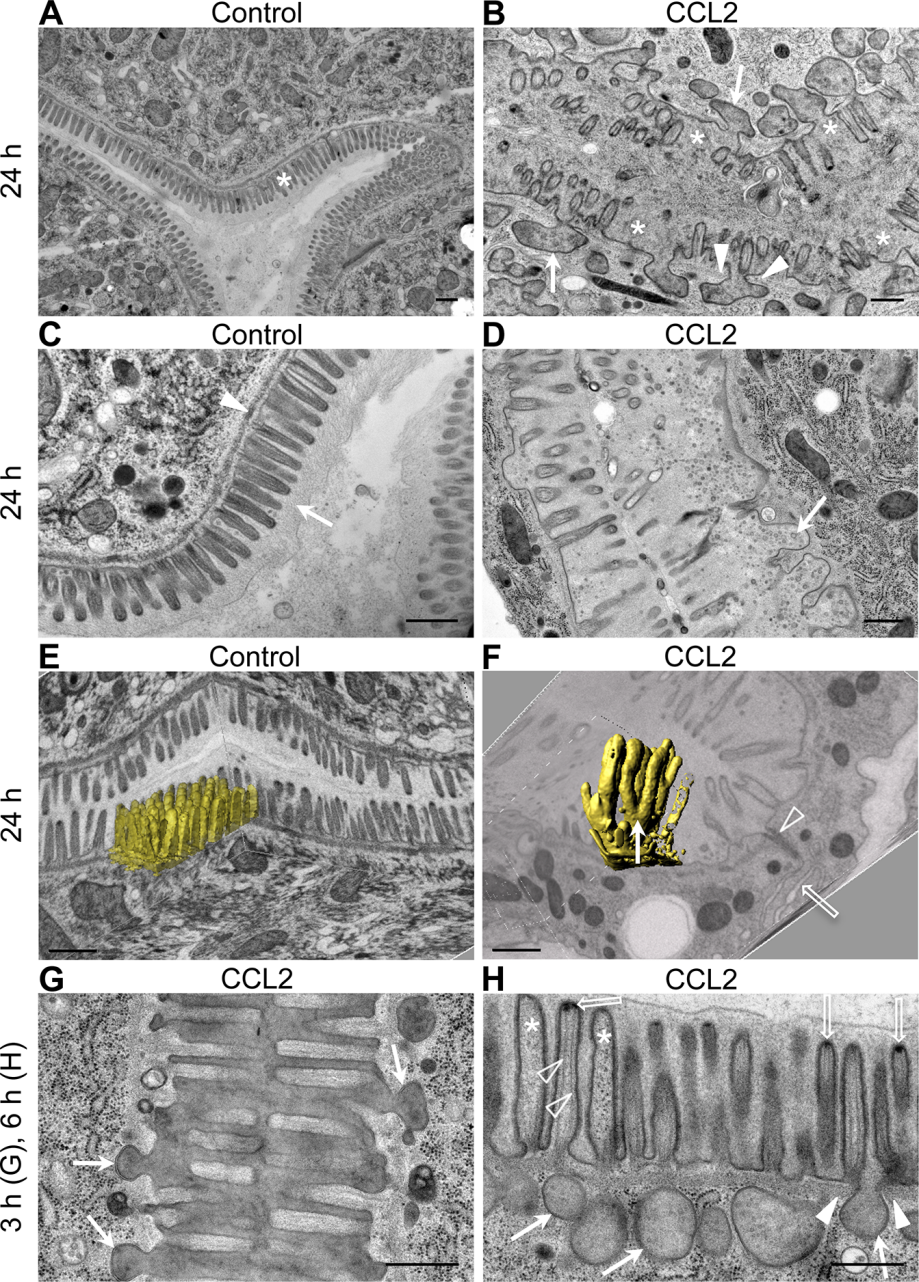
CCL2 leads to microvilli loss, intestinal plasma membrane invaginations, and terminal web gaps. Wild-type *C*. *elegans* L4 larvae were fed on control (empty vector) (A,C,E) or CCL2-expressing *E*. *coli* (B,D,F-H) for the indicated hours and then observed under a transmission (A-D, G,H) or focused ion beam scanning electron microscope (E,F). (A,C) The intestinal apical surface of control animals showed the typical dense brush border made of microvilli (asterisk) and glycocalyx (arrow), and an intact terminal web (arrowhead) underlying the intestinal apical plasma membrane. (B,D) Exposure to CCL2 prompts missing microvilli (asterisks); invaginations of intestinal apical plasma membrane filled with lumenal content (arrows); and disruption of the terminal web (arrowheads). (E,F) 3D reconstruction of selected microvilli in control (E) and CCL2-treated (F) animals showing the disorganized brush border following CCL2 exposure; fused microvilli (yellow; filled arrow); cell border (open arrow); adherens junction (arrowhead). (G,H) Morphological changes are already visible at the ultrastructural level after 3 h (G) and 6 h (H) of exposure to CCL2; invaginations of plasma membrane (filled arrows); terminal web (filled arrowheads); dark cap at the tip of microvilli (open arrows); intact actin filament bundles (open arrowheads); depolymerized actin filament bundles (asterisks). Scale bar: 500 nm.

To analyze the impact of CCL2 binding on the intestinal apical plasma membrane at ultrastructural level, wild-type *C*. *elegans* L4 larvae were analyzed using transmission electron microscopy (TEM) (Fig 3). Control animals showed the typical intestinal brush border morphology (S1B Fig) consisting of a dense brush border of microvilli (Fig 3A, asterisk) surrounded by a glycocalyx (Fig 3C, arrow) and stabilized by the terminal web, a network of actin and intermediate filaments running below and in parallel to the apical plasma membrane ([27]; Fig 3C, arrowhead). By contrast, in animals fed 24 h on CCL2-expressing *E*. *coli*, the intestinal brush border was highly disordered: many microvilli were lost (Fig 3B, asterisks) and the plasma membrane previously surrounding these microvilli formed invaginations into the cytoplasm (Fig 3B, arrows), penetrating through large gaps in the terminal web (Fig 3B, arrowheads). Structures resembling detached microvilli and other debris were visible in the intestinal lumen (Fig 3D, arrow and S2A Fig, open arrows). Unlike the apical surface, the baso-lateral surface between neighboring cells, including the adherens junctions, appeared normal (Fig 3F, open arrow and arrowhead and S1B Fig).

To reconstruct the microvilli in three dimensions, we performed focused ion beam scanning electron microscopy (FIB-SEM) on wild-type L4 larvae fed for 24 h on control (empty vector) or CCL2-expressing *E*. *coli*. In control-fed worms, the microvilli of the dense brush border neatly aligned and were equal in length (Fig 3E and S1 Video). By contrast, the brush border of CCL2-fed animals was highly disorganized. Microvilli also occasionally fused to each other, building coral-reef like structures (Fig 3F, filled arrow and S2 Video).

To determine the kinetics of apical structure destruction, we analyzed animals treated with CCL2-expressing *E*. *coli* for 3 and 6 h. Invaginations were visible already after 3 h (Fig 3G, arrows). After 6 h on CCL2-expressing *E*. *coli*, the invaginations had increased in number and size (Fig 3H, filled arrows) and penetrated through gaps in the terminal web (Fig 3H, filled arrowheads). Often, the invaginations appeared as detached big vesicle-like structures but they were always connected with the plasma membrane at another focal plane (based on FIB-SEM z-stack analysis). In some microvilli, the stabilizing actin filament bundles were depolymerized (Fig 3H, asterisks); these microvilli also lost the dark cap normally visible at their tip (Fig 3H, open arrows and S2B Fig, arrows). These results indicate that a few hours of exposure to CCL2 are sufficient to cause large-scale structural damage to the intestinal brush border.

**Fig 4:**
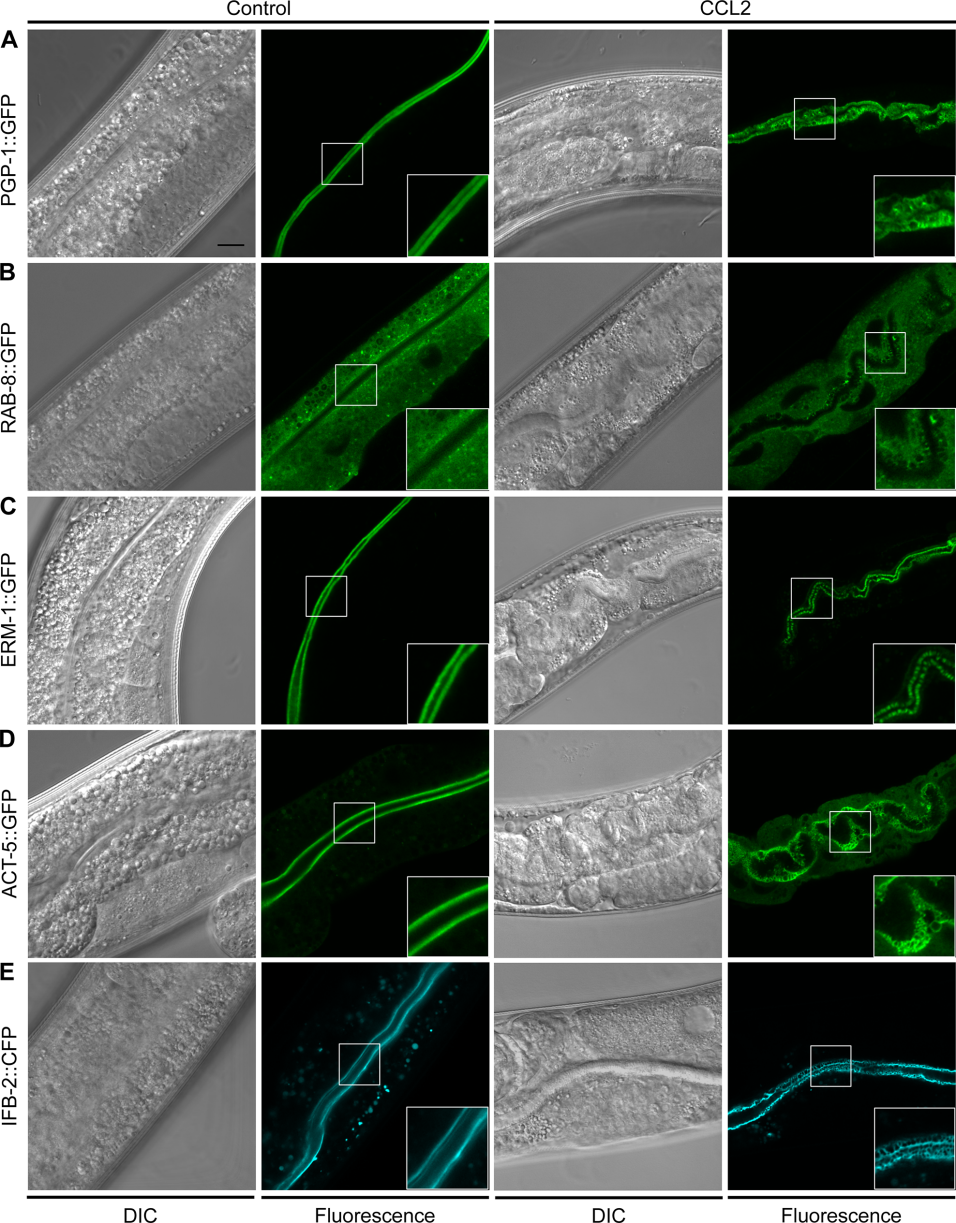
CCL2 alters the structure of the intestinal brush border and terminal web in living animals. (A-E) *C*. *elegans* L4 larvae of the indicated genotype were fed for 24 h on control (empty vector) or CCL2-expressing *E*. *coli*. Various fluorescent transgenic reporters were used to detect changes in intestinal cell architecture by confocal microscopy. (A) PGP-1::GFP is expressed at the intestinal apical plasma membrane. (B) RAB-8::GFP labels the intestinal cytoplasm. (C) ERM-1::GFP links the intestinal apical plasma membrane to the actin filaments within the microvilli. (D) ACT-5::GFP labels actin filaments within the microvilli and the terminal web underlying the intestinal apical plasma membrane. (E) IFB-2::CFP labels intermediate filaments in the terminal web. Scale bar: 10 μm; inset: 2x magnification of the lumenal section of the intestine.

To confirm the structural changes observed by electron microscopy in living animals, we fed CCL2 to *C*. *elegans* strains expressing fluorescent fusion proteins labeling various subcellular structures of the intestine (Fig 4). We first analyzed the effect of CCL2 on the intestinal apical plasma membrane using PGP-1::GFP. While GFP expression at the apical plasma membrane was detectable as a continuous straight line flanking a thin intestinal lumen in control animals, the plasma membrane in CCL2-treated worms was deformed and featured “dark spots” (Fig 4A), which presumably correspond to the invaginations that we observed with electron microscopy (Fig 3). Apical membrane deformations and invaginations could also be observed with a RAB-8::GFP reporter [26], which is involved in protein trafficking and labels the intestinal cytoplasm (Fig 4B and S1B Fig). We followed the loss of microvilli using ERM-1::GFP [28] and ACT-5::GFP [29] reporter strains. The ERM-1 protein links the actin filaments in the microvilli to the intestinal apical plasma membrane (S1B Fig); ACT-5 is the major constituent of the actin filaments found in the microvilli and in the terminal web (S1B Fig). In control animals, the brush border was so dense that at the confocal level both ERM-1 and ACT-5 were visible as thin continuous lines. Upon exposure to CCL2, both the ERM-1::GFP and the ACT-5::GFP lines were frequently interrupted, presumably due to the loss of microvilli (Fig 4C and 4D). To determine the extent of terminal web gaps, we exposed a *C*. *elegans* IFB-2::CFP line [30] to CCL2. IFB-2 is an intermediate filament that is part of the terminal web (S1B Fig). On control *E*. *coli*, the terminal web was visible as two straight, compact, parallel lines framing the intestinal lumen. By contrast, CCL2-treated worms showed many interruptions in these lines (Fig 4E), confirming the presence of gaps in the terminal web.

Taken together, our results show that exposure to CCL2 leads to a deformed intestinal apical plasma membrane, loss of microvilli, and gaps in the underlying terminal web.

### CCL2 does not induce pore formation in the intestinal apical plasma membrane

**Fig 5:**
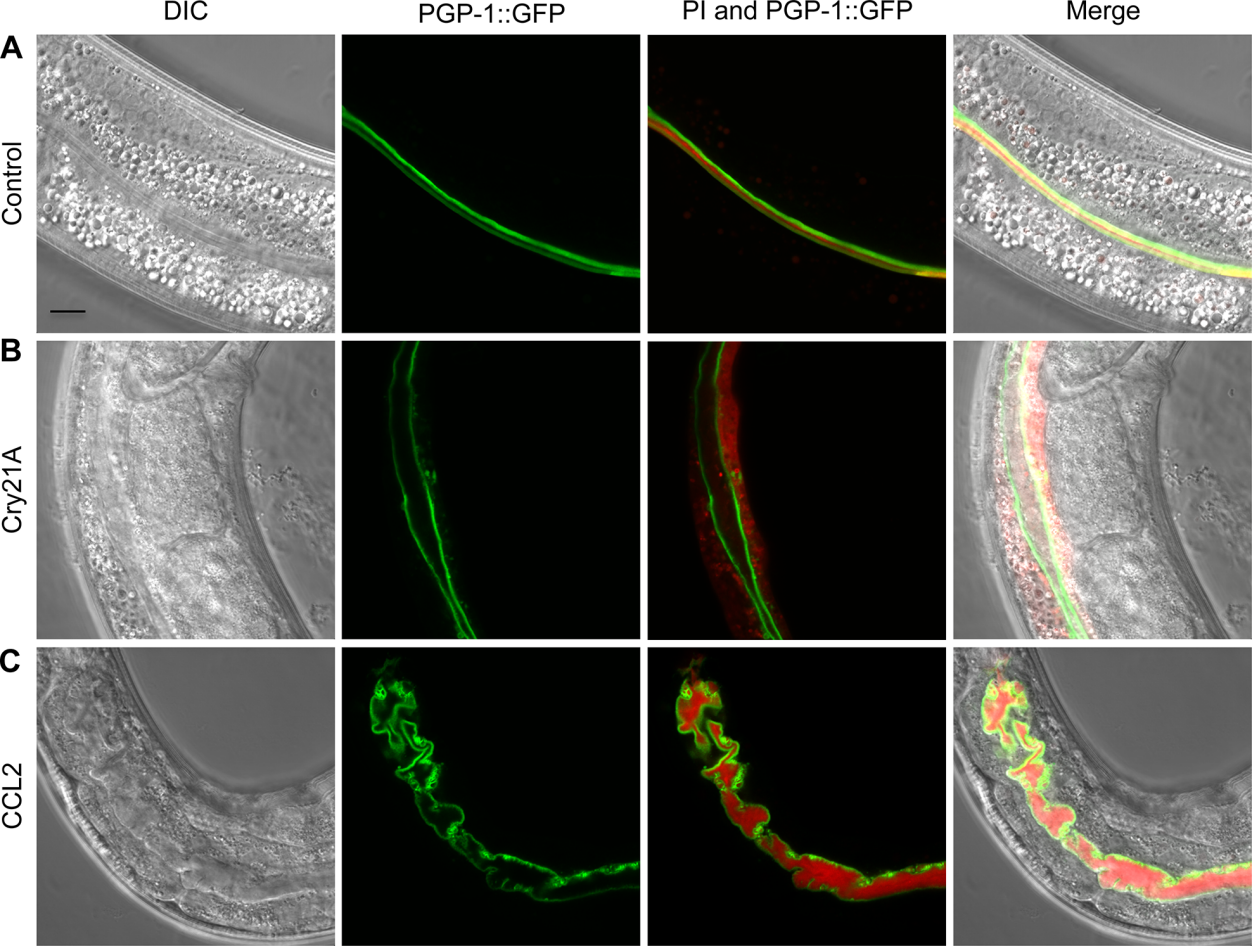
CCL2 does not induce pore formation in the intestinal apical plasma membrane. (A-C) *C*. *elegans* L4 larvae expressing PGP-1::GFP were fed on control (empty vector pQE30) (A), Cry21A- (B) or CCL2- (C) expressing *E*. *coli* for 24 h, transferred into wells containing propidium iodide (PI; red) for 2 h and observed using confocal microscopy. Cry21A is a pore-forming toxin of *B*. *thuringiensis* [31]. PI entered the intestinal cytoplasm of Cry21A-fed (B), but not of control- (A) or CCL2- (C) fed animals. Scale bar: 10 μm.

How does exposure to CCL2 result in the numerous intestinal defects described above? Some toxins, such as members of the Cry protein family of *B*. *thuringiensis*, are known to disrupt intestinal function through generation of pores in the intestinal apical plasma membrane [21]. To test whether CCL2 also induces pore formation, *C*. *elegans* L4 larvae expressing PGP-1::GFP were grown on control (empty vector pQE30), Cry21A- (a known pore-forming toxin [31]) or CCL2-expressing *E*. *coli* for 24 h, followed by a 2 h exposure to propidium iodide (red), which can enter and stain intestinal cells when pores are present in the plasma membrane [32] (Fig 5). In control animals, propidium iodide was restricted to the lumen (Fig 5A), whereas in Cry21A-fed worms, propidium iodide was localized in the cytoplasm, confirming its pore-forming activity (Fig 5B). In CCL2-fed animals, propidium iodide was confined to the intestinal lumen, suggesting that the plasma membrane, in spite of its obvious deformation, maintained its integrity (Fig 5C). That CCL2 and the pore-forming crystal proteins of *B*. *thuringiensis* have distinct molecular modes of action is also suggested by the observation that they induce significantly different sets of morphological defects at the intestinal brush border, both at the cellular and ultrastructural level (Figs 3 and 5 and [32]).

### Exposure to CCL2 leads to a rapid decrease in dipeptides and increase in fatty acids

Morphological signs of toxicity were visible already after 3 h of exposure to CCL2 (Fig 3G). To identify early molecular markers of CCL2 intoxication, and possibly gain insight into to the mechanism of CCL2 action, we performed a metabolomic study on wild-type and on *bre-1(ye4)* (resistant to CCL2 [10]) L4 larvae exposed for 3 h either to wild-type CCL2 or to a carbohydrate-binding deficient, non-toxic CCL2 variant (CCL2M; see Materials and Methods) (S3 Fig). Out of 280 metabolic compounds quantified, 101 (36%) showed a significant change (p≤0.05) in wild-type *C*. *elegans* following exposure to CCL2 (S1 Table), suggesting a dramatic change in the animal’s metabolism. Striking changes included a significant reduction in the abundance of most dipeptides (55/71, 77%; S3A Fig and S1 Table), whereas a large fraction of free fatty acids (10/28, 36%) and lysophospholipids (6/25; 24%) were increased in abundance (S3B Fig and S1 Table). Both changes were specifically associated with CCL2 toxicity, as neither was observed in CCL2-fed *bre-1(ye4)* mutants.

Consistent with our prior phenotypic analyses [10], *bre-1(ye4)* mutants were fully resistant to CCL2 treatment also at a metabolomic level: only 11 out of 280 metabolic compounds showed changes at a significance level of p≤0.05, and none at p≤0.01 (S1 Table). Somewhat surprisingly, *bre-1(ye4)* itself also had only a very weak overall impact on the *C*. *elegans* metabolome. Only 23 compounds showed alterations between the wild type and *bre-1* mutants at a significance level of p≤0.05, and only three at p≤0.01, which is close to the expected false discovery rate (S1 Table).

In summary, our metabolomic analysis shows that exposure to CCL2 rapidly gives rise to extensive changes in intestinal metabolism in wild-type animals, and confirms the full resistance to toxicity conferred by the loss of BRE-1 function.

### CCL2 is not toxic in the absence of bacteria

**Fig 6:**
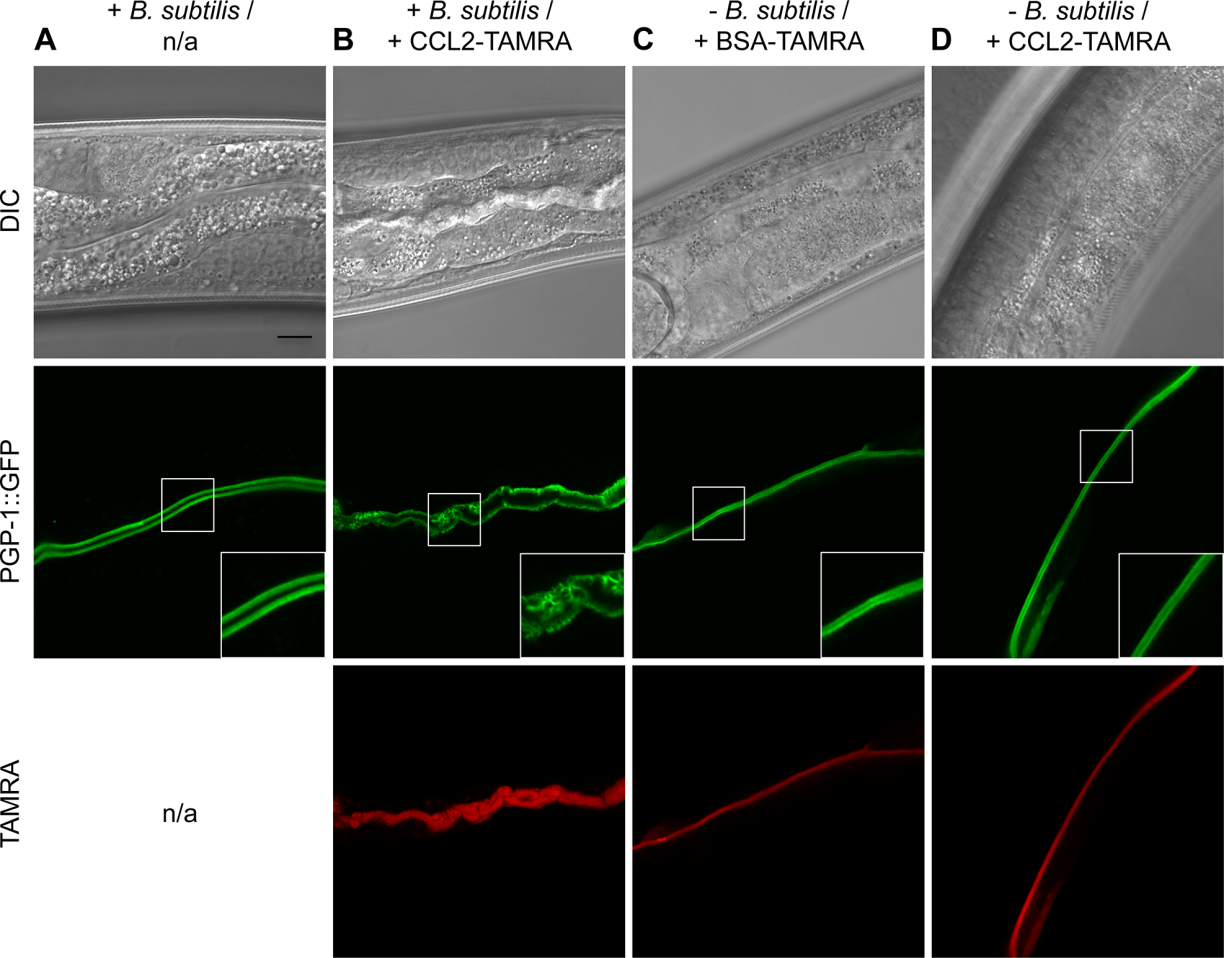
CCL2 is not toxic in the absence of bacteria. (A-D) *C*. *elegans* L4 larvae expressing PGP-1::GFP were exposed to the indicated TAMRA-labeled protein (red; BSA (bovine serum albumin); B-D) for 24 h in the presence (A,B) or absence (C,D) of *B*. *subtilis* and observed using confocal microscopy. Only the combination of CCL2-TAMRA and *B*. *subtilis* led to toxicity (B). Scale bar: 10 μm; inset: 2x magnification of the lumenal section of the intestine; n/a = not applicable.

In all experiments described so far, CCL2 was always administered to *C*. *elegans* together with bacteria as a food source (either *E*. *coli* BL21(DE3) on plates, or *Bacillus subtilis* in liquid culture; see Materials and Methods). Many bacterial species, including *E*. *coli*, are to various degrees toxic to *C*. *elegans* [20]. To find out if bacteria might directly or indirectly contribute to the CCL2 toxicity mechanism, we exposed L4 larvae expressing PGP-1::GFP for 24 h to CCL2 in the presence or absence of bacteria (Fig 6). Whereas simultaneous administration of CCL2-TAMRA and *B*. *subtilis* led to the characteristic intestinal defects described above (Figs 2D and 6B), treatment with CCL2-TAMRA in the absence of bacteria failed to induce any plasma membrane alterations (Fig 6D), suggesting that the presence of bacteria is required for CCL2 toxicity.

Bacteria could contribute directly to CCL2 toxicity, e.g. through synergic or additive damage to the intestinal brush border. Alternatively, bacteria might simply be needed as a food source, e.g. to ensure a high degree of intestinal cell metabolic activity, which might sensitize the intestine to CCL2 action. We tried to distinguish between these two hypotheses by exposing *C*. *elegans* to CCL2 under axenic (bacteria-free) growth conditions (S4 Fig). Exposure of PGP-1::GFP L4 larvae to CCL2-TAMRA for 24 h in axenic medium failed to induce any change in intestinal apical membrane morphology (S4C Fig), indicating that CCL2 is not toxic under these conditions. However, axenic medium is far from optimal as a food source and generally leads to slow growth [33], which might not be sufficient to support CCL2 toxicity. For example, we found that PGP-1::GFP L1 larvae grown for 3 days in axenic medium only reached the L2 stage, whereas they would readily reach L4 stage and adulthood when fed bacteria. L4 larvae fed with axenic medium for 24 h were also slower in their development to adults. It is thus not yet possible to definitively exclude the “bacteria as food” model, and further experiments will be necessary to determine the exact role of bacteria in CCL2-mediated toxicity.

### Reduced expression of ACT-5 induces a phenotype similar to CCL2

We showed above that exposure to CCL2 leads to altered fluorescence patterns of many intestinal apical surface proteins (Fig 4). These changes could simply be markers of CCL2 toxicity. Alternatively, some of the observed phenotypes could be the result of the direct inactivation and / or mislocalization of one or several of these proteins by a CCL2-triggered toxicity pathway. For example, we showed that ACT-5::GFP is relocalized upon exposure to CCL2 (Fig 4D), that some ACT-5 actin filament bundles depolymerized in the microvilli (Fig 3H), and that the actin filament containing terminal web is discontinuous (Fig 3). Thus, interference with ACT-5 function might induce the CCL2 toxicity phenotype in *C*. *elegans*.

To address this issue, we asked whether we could phenocopy the toxicity of CCL2 by performing RNAi-mediated knockdown of *act-5* (S5 Fig). *C*. *elegans* L1 larvae expressing PGP-1::GFP growing on *E*. *coli* expressing *act-5* double-stranded RNA (dsRNA) arrested at the L2 stage. If put as L4 larvae on *E*. *coli* expressing *act-5* dsRNA, animals started showing overt signs of sickness (small, pale adults with poor fertility) after 48 h. By confocal microscopy, changes in the intestinal apical membrane were visible after 24 h. The lining of the intestinal apical plasma membrane was still present but the membrane formed “bubbles” towards the cytoplasm (S5 Fig). Exposure to CCL2 led to a similar adult phenotype but their development to reach adulthood was not arrested, only delayed. At confocal microscopy level, the whole intestinal apical plasma membrane was affected (Fig 4A). We conclude that interference with ACT-5 function might contribute, but cannot be the sole cause of CCL2 toxicity.

### Intestinal expression of α1,3-fucose modified N-glycan cores is sufficient for toxicity of CCL2

**Fig 7:**
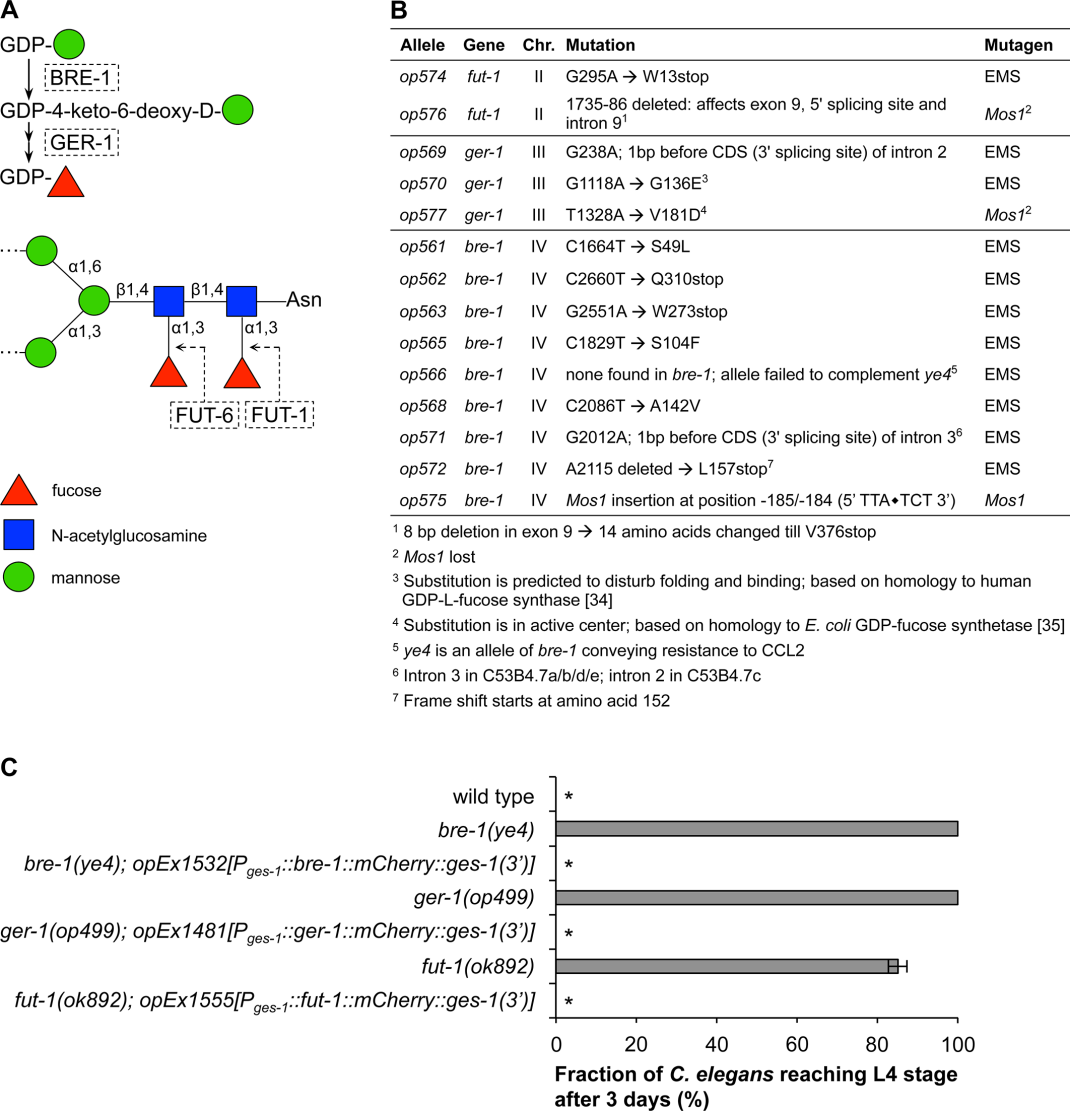
Intestinal expression of α1,3-fucosylated N-glycan cores is sufficient for toxicity of CCL2. (A) Structure of the *C*. *elegans* CCL2 core glycotarget [10,25]. (B) Alleles conferring resistance to CCL2 recovered after transposon (*Mos1)*- or EMS mutagenesis. (Chr.: chromosome; Mutation: base pair change and its position (position 1: “A” of start codon ATG) in the genomic sequence as well as the resulting amino acid change. (C) Intestinal expression of wild-type *bre-1*, *ger-1*, or *fut-1* in the respective *C*. *elegans* mutant was sufficient to restore sensitivity to CCL2. All constructs were under the control of the intestinal promoter *ges-1* and C-terminally tagged with *mCherry* (red). Transgenic and control animals were grown on CCL2-expressing *E*. *coli* for 3 days. Data shown are average ± standard error of the mean of three experiments. Asterisks (*): After 3 days, all worms were L2 larvae.

We showed above that binding of CCL2 to the intestinal apical surface results in toxicity (Fig 2). We previously demonstrated that CCL2 recognizes a fucosylated N-glycan core structure ([10,25]; Fig 7A). It is thus likely that CCL2 induces toxicity through its binding to one or several proteins that carry this sugar structure and that are localized on the intestinal apical surface. In an effort to identify this / these protein(s), or any other molecule involved in CCL2-mediated toxicity, we performed mutagenesis screens with the transposon *Mos1* and with the chemical ethyl methanesulfonate (EMS) on wild-type *C*. *elegans*. F2 progenies of randomly mutagenized worms were let to hatch on plates seeded with CCL2-expressing *E*. *coli* and resistant mutants were recovered. All fourteen isolated mutations affected the previously identified resistance genes *bre-1* (9 alleles), *ger-1* (3 alleles; [34,35]), or *fut-1* (2 alleles) (Fig 7B). These results confirm the importance of the proximal α1,3-fucose on the N-glycan cores for CCL2 toxicity.

Since CCL2 binds its glycotarget at the apical surface of the intestine (Fig 2), we wanted to test if expression of *bre-1*, *ger-1*, and *fut-1* in the intestine alone would be sufficient to confer sensitivity to CCL2. To this aim, we constructed transgenic *C*. *elegans* strains expressing the three wild-type genes in the intestine only (using the gut-specific *ges-1* promoter) in the respective mutant background (*bre-1(ye4)*; *ger-1(op499)*; *fut-1(ok892)*). As expected, all three transgenes restored sensitivity to CCL2 (Fig 7C).

Taken together, our data indicate that interaction between CCL2 and its fucosylated glycotarget, which is found on one or several proteins present on the intestinal apical surface, is sufficient to induce a broad range of morphological and functional defects ultimately resulting in premature death.

## Discussion

In this work, we describe the detailed pathology induced in *C*. *elegans* by CCL2, a fungal lectin that causes toxicity solely through its ability to bind to its glycotarget(s) on the intestinal apical surface. Exposure to CCL2 leads to loss of microvilli, shedding of microvilli fragments into the lumen, actin depolymerization, and invaginations of the plasma membrane previously surrounding the microvilli through gaps in the terminal web. Overall, the intestine of *C*. *elegans* is faced with a destabilized and fragmented brush border and terminal web, resulting in greatly disturbed digestion / nutrient uptake, and consequently delayed development, reduced brood size, and premature death.

What is the mechanism of CCL2 toxicity? Our experiments ruled out many of the known ways in which lectins can cause cellular damage. The 15 kDa CCL2 consists only of a single carbohydrate-binding domain and has no known enzymatic activity [10], is not internalized by intestinal cells (Fig 2), and does not induce pore formation (Fig 5). There were also no signs of endocytosis of the PGP-1::GFP stained plasma membrane (Fig 4A) as can be observed following intoxication with pore-forming toxins [32]. Finally, there was no loss in intestinal polarity upon exposure to CCL2, since the transmembrane protein PGP-1::GFP was still exclusively expressed on the apical surface of the intestine after 24 h (Fig 4A). Since several toxicity mechanisms could be excluded for CCL2, we propose that CCL2 employs a novel mode of action.

Exposure to CCL2 highly disturbed the metabolism of *C*. *elegans*. The most striking changes were a reduction in the abundance of dipeptides and an increase in fatty acids. Dipeptides are produced in the intestinal lumen by hydrolysis of bacterial proteins and taken up by *C*. *elegans* intestinal cells via e.g. the PEPT/OPT H+-coupled oligopeptide transporter family [36,37]. A reduction in overall dipeptide abundance could be the result of a disturbed intestinal function–either from a decreased degradation of proteins into dipeptides in the intestinal lumen, or from decreased dipeptide uptake into the intestinal cells, prompting their eventual loss through defecation. The increase in free fatty acids and in lysophospholipids could be the result of either altered degradation or uptake of bacterial lipids, or of an increased degradation of endogenous *C*. *elegans* lipids. We consider the latter hypothesis more likely, as we also observed increased concentrations of lysophospholipids deriving from phosphatidylcholine (2/8) and phosphatidylinositol (2/4) (together: 4/12; 33%; S1 Table), which are not found in bacteria and thus must be of *C*. *elegans* origin [38]. The increased phospholipid degradation might be an early sign of the large-scale plasma membrane loss that is observable a few hours later.

Interestingly, bacteria are required for CCL2 to be toxic (Fig 6). A similar requirement has previously been reported for *B*. *thuringiensis*-mediated insecticidal activity and for dietary lectins [39,40], whereas e.g. *Pseudomonas aeruginosa* PA14 produces diffusible toxins that are also toxic in the absence of bacteria [41]. The contribution of bacteria to CCL2 toxicity is unclear. Both gram-negative (*E*. *coli*) and gram-positive bacteria (*B*. *subtilis*) enable CCL2 toxicity, suggesting a general or evolutionary conserved mechanism. Live bacteria in the intestine of *C*. *elegans* are not required for CCL2 toxicity since no intact bacteria were observed in the intestinal lumen. Bacterial enzymes or other cellular components might be actively involved in CCL2 toxicity. They might be needed to unmask the CCL2 glycotarget [42] or to damage the plasma membrane. Indeed, Miyake et al. reported that lectins were only toxic to cell cultures upon artificial membrane damage [43]. A functional plasma membrane repair mechanism and an active immune system have to be in place to fight naturally occurring damage by intestinal bacteria [43–45]. CCL2 could negatively regulate either of them, e.g. by hindering exocytosis of new membrane patches or repolymerization of actin filaments or by blocking receptors of endogenous lectins involved in innate immunity [43,44,46]. Alternatively, bacterial components could have a mere passive function in CCL2 toxicity, e.g. by stimulating endo- and exocytosis as part of digestion, which might lead, together with the action of CCL2, to a loss of homeostasis at the intestinal plasma membrane. In any case, CCL2 does not seem to be a primary “toxin”.

How does CCL2 act at the molecular level? The binding of CCL2 to protein targets in the glycocalyx or on the intestinal apical plasma membrane could prompt the activation or inactivation of (a) signaling pathway(s) that then disrupt(s) directly or indirectly the structural integrity of the brush border. The fact that the *bre-1*, *ger-1*, and *fut-1 C*. *elegans* mutants are healthy despite the lack of GDP-fucose [47] suggests either that CCL2 does not inhibit a pathway, or that the pathway inhibited by CCL2 can be activated by its endogenous ligand independent of fucose-binding. Rather than affecting a signaling pathway, CCL2 might, through binding to its target, interfere with and disturb the network of glycoproteins that make up the glycocalyx [48]. This in turn might destabilize the microvilli embedded in the glycocalyx and result in their progressive loss. In addition, or as an alternative, disturbance of the glycocalyx network could allow bacterial or endogenous enzymes access to the plasma membrane and damage it [7].

One way for CCL2 to exert its toxicity might be by affecting the function of proteins that are compounds of microvilli or the terminal web (S1B Fig). Knockdown or knockout of the genes coding for these proteins might thus potentially phenocopy CCL2 toxicity. Even though knockdown or knockout of *act-5*, *eps-8*, *erm-1*, and several intermediate filaments do affect brush border and terminal web structures [27,30,49–51], none of their phenotypes at the fluorescence or TEM microscopy level was similar to what we observed with CCL2, with the exception of knockdown of *act-5*, which can induce terminal web gaps [19]. However, knockdown of *act-5* on PGP-1::GFP expressing worms only partially reproduced the plasma membrane changes observed following CCL2 treatment (S5 Fig and Fig 4A). Thus, interference with ACT-5 function might contribute to the CCL2 toxicity pattern but it seems unlikely to be the sole cause.

To identify the protein(s) carrying the glycotarget and / or additional components involved in CCL2 toxicity, we performed several genetic screens for *C*. *elegans* mutants resistant to CCL2 toxicity. However, the only resistance genes that we could identify were the previously characterized enzymes involved in the production and addition of the proximal fucose onto the N-glycan core (Fig 7A and 7B). This suggests that we have saturated the universe of single genes that confer, through simple loss of function, resistance to CCL2 without interfering with animal viability. We conclude that the CCL2 targets, as well as other proteins involved in the toxicity mechanism, are likely either redundant or essential. Other approaches, such as biochemical purification of CCL2-interacting proteins, will thus be required to identify the proteins directly targeted by CCL2.

Previous studies have characterized the effect of various bacterial and microsporidial pathogens as well as lectins on the intestine by TEM [19,22,32,40]. Although some of these also led to loss of microvilli, the overall phenotype of the brush border was always clearly different from the one after CCL2 exposure. Strikingly, and by contrast, the CCL2 phenotype is quite similar to the one observed in mammalian brush border cells following exposure to the dietary lectins WGA or Con A, which also lead to depolymerization of microvilli and invaginations of the intestinal plasma membrane [9]. That lectins can elicit similar cellular pathologies in species as distant as worms and mammals suggests that despite the considerable difference in gross anatomy, the cellular anatomy and physiology of the intestinal brush border is highly conserved between *C*. *elegans* and mammals. Thus, *C*. *elegans* might be a promising model organism to study pathologies, such as exposure to dietary lectins, that affect intestinal brush border function in mammals.

## Materials and Methods

### Strains

*C*. *elegans* and bacterial strains used in this study are listed in S2 Table.

### Recombinant and purified CCL2 from *C*. *cinerea*

CCL2 isolation, purification, and expression in the pet24b vector (Novagen, Merck KGaA, Darmstadt, Germany) in *E*. *coli* BL21(DE3), as well as generation and purification of His8-tagged CCL2 was performed as described [10]. CCL2 and BSA were TAMRA-labeled (Molecular Probes, Life Technologies, Carlsbad, USA) as described [52].

### *C*. *elegans* culturing conditions

All *C*. *elegans* strains were maintained at 20°C on nematode growth media (NGM) agar plates seeded with *E*. *coli* OP50 [53] with the following exceptions: *E*. *coli* BL21(DE3) was used to express recombinant CCL2; *E*. *coli* JM109(DE) was used to express Cry21A; *E*. *coli* HT115(DE3) was used to express dsRNA for RNAi; *B*. *subtilis* was used for liquid cultures.

### Toxicity assays on plates with unlabeled recombinant CCL2

NGM agar plates containing isopropyl β-D-1-thiogalactopyranoside (IPTG; 2 mM) to induce expression of the bacterial plasmid, and kanamycin (0.1 mM) to avoid bacterial contamination, were seeded with an overnight culture of *E*. *coli* BL21(DE3) strains (Novagen) containing the pet24b plasmid with CCL2 (toxicity) or without CCL2 (empty vector; control) in lysogeny broth (LB) [53]. As positive control for the pore-forming assay, the pore-forming toxin Cry21A (crystal protein) of *B*. *thuringiensis* [31] and its empty vector pQE30 expressed in *E*. *coli* JM109(DE3) were grown on NGM agar plates containing IPTG (1 mM) and ampicillin (0.3 mM).

For the developmental assay, adult *C*. *elegans* worms were bleached [53] and the embryos left to hatch on plates seeded with the appropriate bacteria (day 1). After 44 h, images were taken or the number of worms reaching the L4 larval stage was determined. For the L4 assay, *C*. *elegans* L4 larvae were grown for 24 h on plates seeded with the appropriate bacteria, followed by microscopic analysis. For the pore-forming assay, the worms were transferred, prior to analysis, for 2 h to 96-well plates containing propidium iodide (6.7 μg/ml) and S-medium only [32,53].

### Toxicity assays in liquid medium with purified CCL2-TAMRA

All assays were done in S-medium in a total volume of 50 μl per well in 96-well plates. Approximately 15 *C*. *elegans* L4 larvae and, if indicated, 5 μl of a bacterial overnight culture concentrated to OD_600_ = 20, and purified CCL2-TAMRA or BSA-TAMRA were added to each well. For the CCL2 binding assay, the worms were incubated with non-toxic concentrations of CCL2-TAMRA (100 μg/ml) and *E*. *coli* BL21(DE3) empty vector as food. After 24 h, the worms were let to crawl on plates seeded with *E*. *coli* BL21(DE3) empty vector for 2 h to wash out unbound CCL2-TAMRA. For the internalization assay, the worms were incubated with toxic concentrations of CCL2-TAMRA (500 μg/ml) and *B*. *subtilis* as food because unlike *E*. *coli* this species does not agglutinate in the presence of high concentrations (500 μg/ml) of purified CCL2. After 1, 3, or 24 h, worms were transferred onto *B*. *subtilis*-seeded plates for 1 h to remove unbound CCL2-TAMRA, followed by microscopic analysis. For the toxicity assay without bacteria, worms grown to the L4 stage on *E*. *coli* OP50-seeded plates were washed 6 times with M9 buffer [53] to remove the bacteria. Approximately 15 L4 larvae / 20 μl S-medium (M9 buffer was replaced by S-medium) were transferred into each well. The worms were incubated for 24 h in wells containing *B*. *subtilis* or no bacteria and / or CCL2-TAMRA (500 μg/ml) or BSA-TAMRA (500 μg/ml). For the toxicity assay with axenic medium, the axenic medium was derived from Castelein et al., 2008 [54] and contained: S-medium; 2.4% (w/v) soy-peptone; 2.4% (w/v) dry yeast extract; 0.5 mg/ml hemoglobin; 5 μg/ml cholesterol; 0.1 mg/ml ampicillin. The worms were incubated for 24 h in wells containing axenic medium or *B*. *subtilis* and / or CCL2-TAMRA (500 μg/ml). All assays were analyzed by confocal microscopy.

### Microscopy of *C*. *elegans*

Dissection microscopy images were taken with a stereo microscope Leica MZ 12.5 equipped with a Nikon Coolpix 990 digital camera. For DIC and fluorescence microscopy, worms were placed on 3% agarose pads in a drop of 10 mM levamisole for anesthesia and mounted under a coverslip. DIC images were taken using a Leica DM6000B microscope equipped with DIC (Nomarski) optics, a PL Fluotar 40x/1.00–0.50NA Oil objective, a Leica DFC360FX camera, and the LAS AF Leica Application Suite software (version 2.6.0.7266; Leica Microsystems, Vienna, Austria). Fluorescence images were taken using a Carl Zeiss LSM710 confocal microscope equipped with a Plan-Apochromat 63x/1.40NA Oil DIC M27 objective and the Zen2009 software (Zeiss, Oberkochen, Germany). Images were false-colored by ImageJ. For transmission electron microscopy (TEM) and focused-ion beam scanning electron microscopy (FIB-SEM), worms were frozen in a HPM 100 high-pressure freezing machine (Leica Microsystems). After freeze-substitution, samples were block stained with 1% uranyl acetate in acetone (stock solution: 20% in methanol) for 1 h at 4°C and embedded in Epon / Araldite (Sigma-Aldrich, St. Louis, USA). Thin sections were post-stained with Reynolds lead citrate and analyzed at 80 kV acceleration voltage using a CM100 transmission electron microscope (FEI, Eindhoven, The Netherlands) equipped with a side mounted digital camera Orius 1000 (Gatan, Munich, Germany). For FIB-SEM, ion milling with an advance of 5 nm per image and image acquisition were performed simultaneously in an Auriga 40 Crossbeam system (Zeiss, Oberkochen, Germany) using the FIBICS Nanopatterning engine (Fibics Inc., Ottawa, Canada). SEM images were acquired at 1.7 kV using an in-lens energy selective backscattered electron detector (ESB) with a grid voltage of 1.4 kV, and a dwell time of 35 or 40 μs. The pixel size was set to 5 nm and tilt-corrected to obtain isotropic voxels. Alignment of the image stack was performed with the Sift plugin [55] of the ImageJ image-processing package [56]. Segmentation of microvilli was done with Imaris (Bitplane AG, Zurich, Switzerland) using the surpass surface tool. For more details, consult the supporting information (S1 Text).

### Metabolomics

Wild-type and *bre-1(ye4) C*. *elegans* L4 larvae were transferred for 3 h to plates seeded with *E*. *coli* expressing wild-type CCL2 or mutated, non-toxic CCL2 (CCL2M; amino acid (aa) change in carbohydrate-binding part: G230A(bp) → G77E(aa)) as negative control; created by PCR-based site-directed mutagenesis (Thermo Scientific, Thermo Fisher Scientific, Waltham, USA)). The worms were collected with phosphate-buffered saline (PBS) buffer, immediately frozen in liquid nitrogen, and subsequently analyzed by Metabolon (www.metabolon.com, Durham, USA). Samples were extracted and prepared for analysis using Metabolon’s standard solvent extraction method. The extracted samples were split into equal parts for analysis on the gas chromatography–mass spectrometry (GC-MS) and liquid chromatography–tandem mass spectrometry (LC-MS/MS) platforms. Also included were several technical replicate samples created from a homogeneous pool containing a small amount of all study samples (“Client Matrix”). Metabolon’s general platform methods are described in [57]. Statistical terminology: q-values: to account for the multiple testing problem, the p-values were adjusted using an optimized false discovery rate approach [58].

### RNA interference (RNAi) assay

Feeding RNAi bacteria was performed as described [59]. In short, *E*. *coli* HT115(DE3) [60] expressing *act-5* dsRNA from vector L4440 (Ahringer RNAi library) were incubated overnight in LB containing ampicillin (0.6 mM) and tetracycline (0.03 mM) and then seeded on NGM agar plates containing ampicillin (0.6 mM) and IPTG (2 mM). Worms expressing PGP-1::GFP were either let to hatch or transferred as L4 larvae onto the RNAi plates (day 1). In both cases worms were followed for 6 days.

### Screen for CCL2 resistant *C*. *elegans* strains by EMS and *Mos1* mutagenesis

EMS (ethyl methanesulfonate) mutagenesis was performed with wild-type *C*. *elegans* (N2) as described [61]. Approximately 40,000 haploid genomes were screened. Adult F_1_ progeny of EMS treated P0 were bleached [53] and distributed onto CCL2-expressing *E*. *coli* plates, and the F_2_ generation was screened for CCL2-resistant worms. Mutations were mapped to chromosomes using fragment-length polymorphisms (FLP) mapping Tier 1 [62]. As all mutations mapped to chromosomes II, III, and IV, which also carry the *fut-1*, *ger-1*, and *bre-1* genes, respectively, we PCR amplified and sequenced the genome locus (exons and introns) of the relevant candidate gene for each mutant. For *op570*, the mutation was only found after whole genome sequencing, which was prepared and performed according to [63]. For six mutations (*op561*, *op565*, *op566*, *op568*, *op570*, *op577*), complementation tests [64] and FLP mapping Tier 2 were performed to increase the confidence that the mutation found by sequencing is responsible for the resistance phenotype.

The *Mos1* transposon insertional mutagenesis was performed with *C*. *elegans* strains mutated in *pmk-1(km25)* as described [65]. Approximately 200,000 haploid genomes were screened. Two of the three recovered resistant mutants lost the *Mos1* transposon but remained resistant as the loss of *Mos1* left a footprint behind, which was identified analogously to the EMS mutants.

### Construction of transgenic worms

The 5’ (1504bp upstream of ATG) and 3’ (1989bp downstream of TAA) regulatory regions of *ges-1* were used to drive the intestinal expression of wild-type *bre-1*, *ger-1*, and *fut-1*. For all three genes, the genomic wild-type sequence from the start codon to one codon before the stop codon were fused to *mCherry* (red) at their 3’ end by conventional restriction digest and ligation. The three parts (5’ and 3’ regions of *ges-1* and gene fused to *mCherry*) were fused by Gateway recombination (Life Technologies, Carlsbad, USA) and cloned into competent bacteria for sequencing and DNA extraction. The transgenic worms were created by micro particle bombardment [66] and crossed into the respective mutant strain *(bre-1(ye4)*; *ger-1(op499)*; *fut-1(ok892))*.

### Accession numbers

Accession numbers for *C*. *elegans* genes and gene products mentioned in this paper are based on Wormbase (http://www.wormbase.org). *pgp-1* (K08E7.9), *rab-8* (D1037.4), *erm-1* (C01G8.5), *act-5* (T25C8.2), *ifb-2* (F10C1.7), *bre-1* (C53B4.7), *ger-1* (R01H2.5), *fut-1* (K08F8.3), *fut-6* (T05A7.5), *ges-1* (R12A1.4). The complementary DNA (cDNA) sequence of CCL2 from *C*. *cinerea* strain AmutBmut was deposited in GenBank (accession number ACD88750).

## Supporting Information

S1 Fig Schematic representation of *C. elegans* and an intestinal cell. (A) Scheme of an adult *C*. *elegans*. Figure modified from Kaletta and Hengartner, 2006 [67]. The intestine of *C*. *elegans* has only 20 non-renewable polarized epithelial cells that form the intestinal tube in nine rings of two directly apposed cells, except for the first ring that is formed by four cells [7]. (B) Scheme of an intestinal cell. The brush border consists of microvilli and a glycocalyx that covers the apical surface of intestinal cells. Microvilli are finger-like protrusions of the intestinal plasma membrane that increase the absorptive surface. Figure modified from McGhee [7].
(TIF)

S2 Fig Microvillar debris in the intestinal lumen after exposure to CCL2. Wild-type *C*. *elegans* L4 larvae were fed on CCL2-expressing (A) or control (B) *E*. *coli* for 24 h and observed under a transmission electron microscope. (A) The intestinal lumen is filled with debris. On one side of the brush border, the microvilli are cut in cross-section (filled arrow). Similar structures, possibly microvillar remnants, can be observed floating in the lumen (open arrows). (B) Dark cap (arrows) and actin filament bundles (arrowheads) are visible in intact microvilli. Scale bar: 500 nm.
(TIF)

S3 Fig CCL2 results in decreased dipeptides and increased free fatty acids. Wild-type (wt) and *bre-1(ye4)* (*bre-1*; resistant to CCL2 [10]) *C*. *elegans* L4 larvae were fed on control (mutated, non-toxic CCL2 (CCL2(G77E) = CCL2M)) or wild-type CCL2-expressing *E*. *coli* for 3 h and were thereafter checked for changes in the abundance of various metabolites. CCL2 treatment led to a decrease in dipeptide (A) and an increase in free fatty acid (B) concentrations. Only two representative molecules are shown for each group.
(TIF)

S4 Fig CCL2 is not toxic to worms fed on axenic medium. (A-C) *C*. *elegans* L4 larvae expressing PGP-1::GFP were fed with axenic medium (A, C) or *B*. *subtilis* (B) together with CCL2-TAMRA (red) (B, C) and observed using confocal microscopy. Only the combination of CCL2-TAMRA and *B*. *subtilis* induced toxicity (B). Scale bar: 10 μm; inset: 2x magnification of the lumenal section of the intestine; n/a = not applicable.
(TIF)

S5 Fig *act-5(RNAi)* also alters the intestinal apical plasma membrane. *C*. *elegans* L4 larvae expressing PGP-1::GFP were fed for 24 h with *E*. *coli* expressing *act-5* dsRNA. Reduced ACT-5 abundance gave rise to a disturbed intestinal apical plasma membrane, in which the lining was still visible but “bubbles” were formed towards the cytoplasm. Two representative images are shown. Scale bar: 10 μm; inset: 2x magnification of the lumenal section of the intestine.
(TIF)

S1 Table Metabolomic profile following CCL2 treatment. *Mean values*: Black-bolded text indicates samples chosen for S3 Fig. *Fold change*: Molecules showing a statistically significant change (p≤0.05) are highlighted: increased abundance, red; decreased abundance, green. q-values (false discovery rate adjusted p-values) give estimates of the false discovery rate. All data are normalized to Bradford protein concentration values.
(XLSX)

S2 Table Bacterial and *C. elegans* strains used in this study. (DOCX)

S1 Text Transmission and FIB scanning electron microscopy. Detailed protocol.
(DOCX)

S1 Video FIB-SEM z-stack and 3D reconstruction of selected microvilli. Exposure of wild-type L4 larvae to control *E*. *coli* for 24 h.
(MP4)

S2 Video FIB-SEM z-stack and 3D reconstruction of selected microvilli. Exposure of wild-type L4 larvae to CCL2-expressing *E*. *coli* for 24 h.
(M4V)
