# Supplementary figures and images for: Disruption of the C. elegans Intestinal Brush Border by the Fungal Lectin CCL2 Phenocopies Dietary Lectin Toxicity in Mammals
Source: PLoS One. 2015 Jun 9;10(6):e0129381. doi: 10.1371/journal.pone.0129381 (PMC4461262; doi:10.1371/journal.pone.0129381)

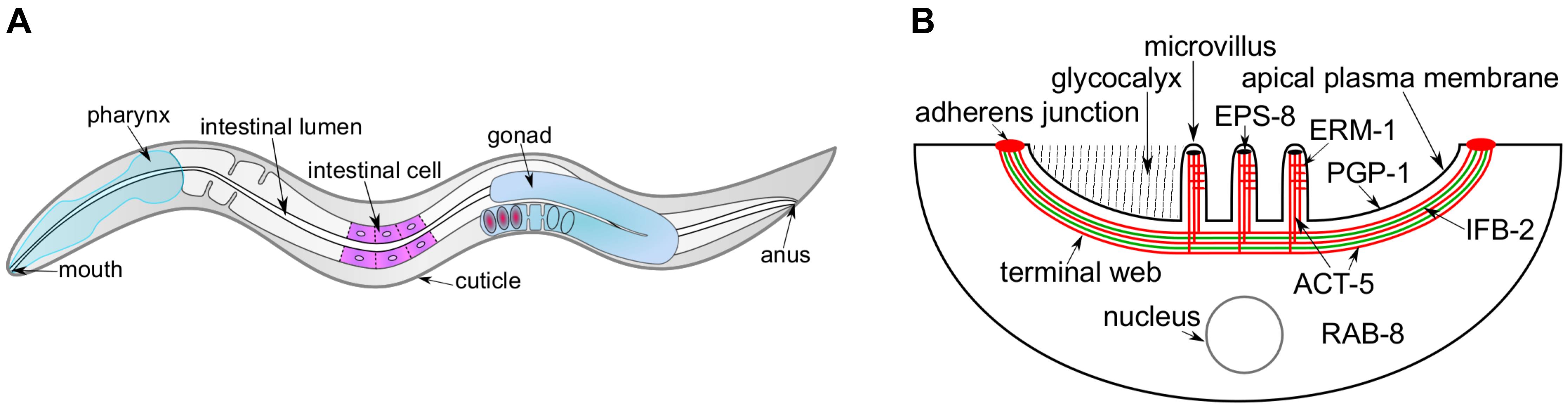

Supplement: S1 Fig — (A) Scheme of an adult C. elegans. Figure modified from Kaletta and Hengartner, 2006 [67]. The intestine of C. elegans has only 20 non-renewable polarized epithelial cells that form the intestinal tube in nine rings of two directly apposed cells, except for the first ring that is formed by four cells [7]. (B) Scheme of an intestinal cell. The brush border consists of microvilli and a glycocalyx that covers the apical surface of intestinal cells. Microvilli are finger-like protrusions of the intestinal plasma membrane that increase the absorptive surface. Figure modified from McGhee [7]. (TIF) [file pone.0129381.s001.tif]

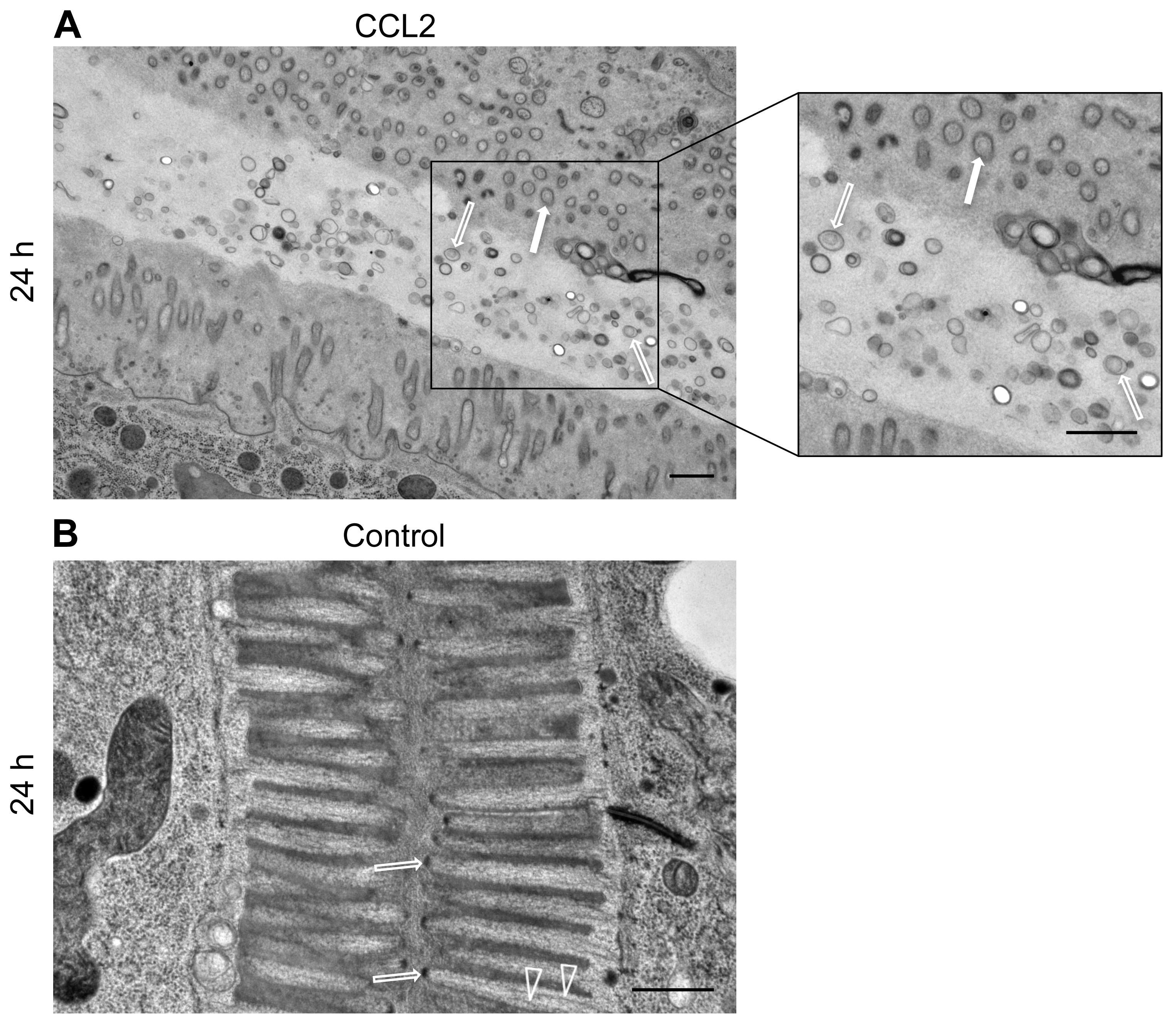

Supplement: S2 Fig — Wild-type C. elegans L4 larvae were fed on CCL2-expressing (A) or control (B) E. coli for 24 h and observed under a transmission electron microscope. (A) The intestinal lumen is filled with debris. On one side of the brush border, the microvilli are cut in cross-section (filled arrow). Similar structures, possibly microvillar remnants, can be observed floating in the lumen (open arrows). (B) Dark cap (arrows) and actin filament bundles (arrowheads) are visible in intact microvilli. Scale bar: 500 nm. (TIF) [file pone.0129381.s002.tif]

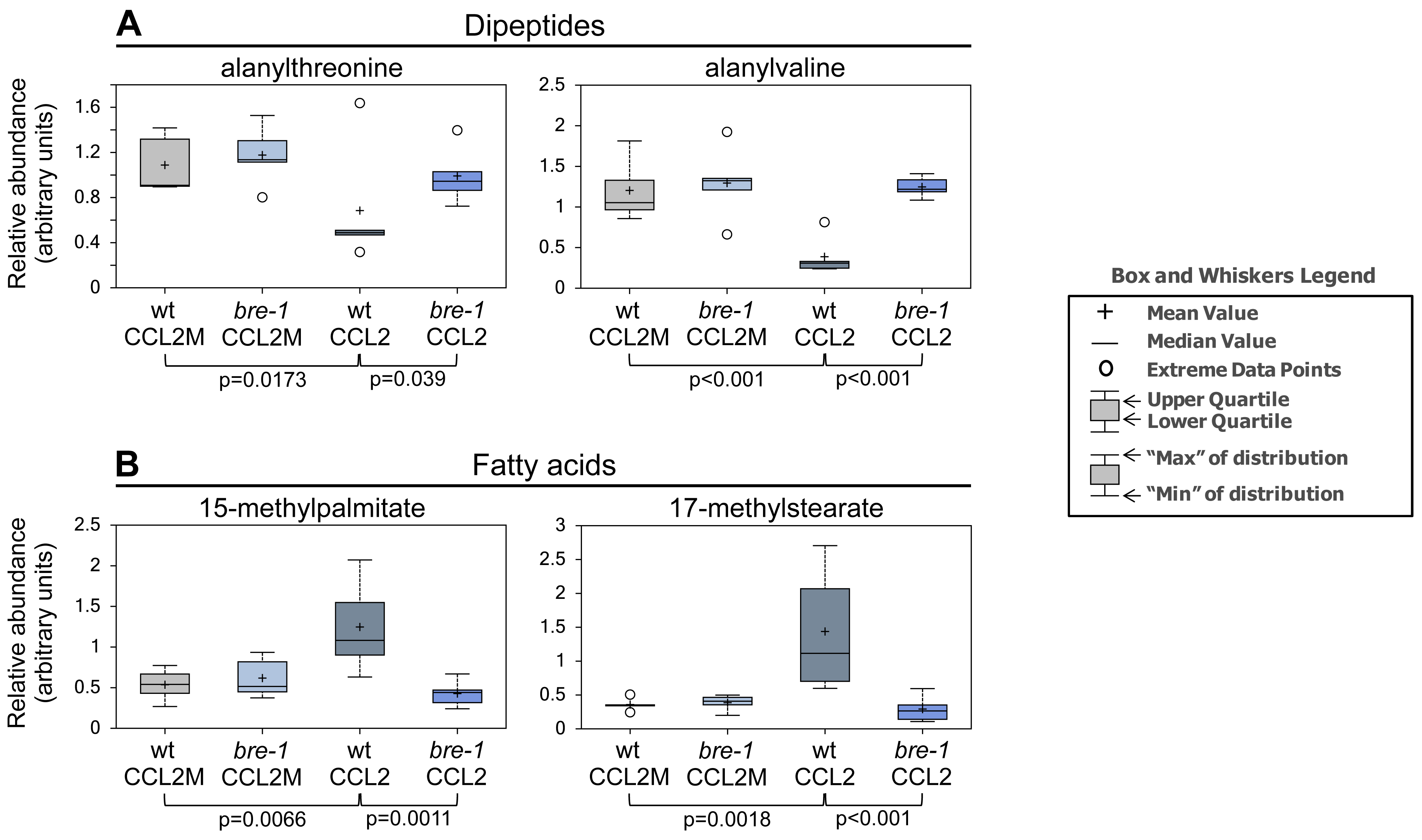

Supplement: S3 Fig — Wild-type (wt) and bre-1(ye4) (bre-1; resistant to CCL2 [10]) C. elegans L4 larvae were fed on control (mutated, non-toxic CCL2 (CCL2(G77E) = CCL2M)) or wild-type CCL2-expressing E. coli for 3 h and were thereafter checked for changes in the abundance of various metabolites. CCL2 treatment led to a decrease in dipeptide (A) and an increase in free fatty acid (B) concentrations. Only two representative molecules are shown for each group. (TIF) [file pone.0129381.s003.tif]

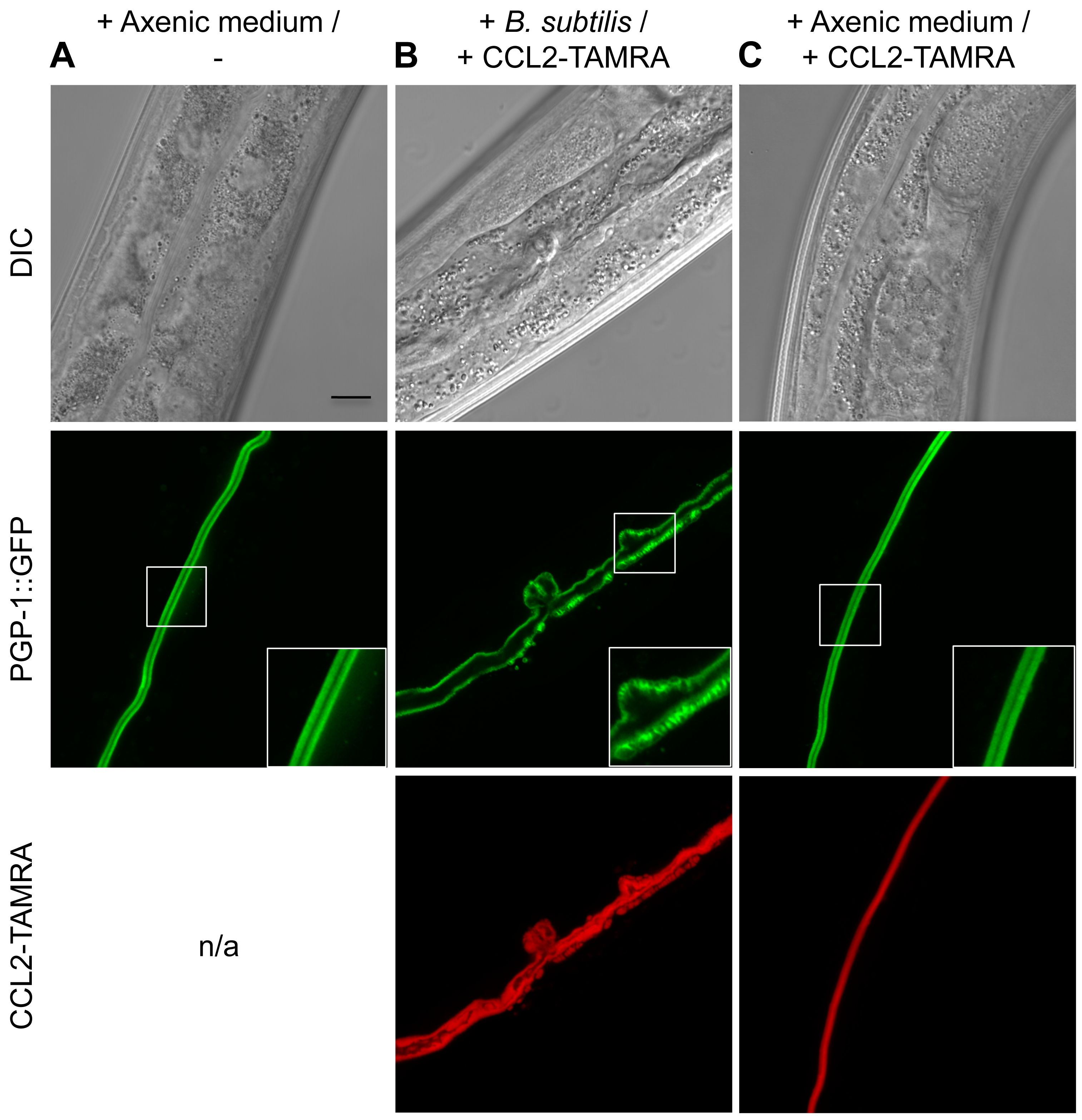

Supplement: S4 Fig — (A-C) C. elegans L4 larvae expressing PGP-1::GFP were fed with axenic medium (A, C) or B. subtilis (B) together with CCL2-TAMRA (red) (B, C) and observed using confocal microscopy. Only the combination of CCL2-TAMRA and B. subtilis induced toxicity (B). Scale bar: 10 μm; inset: 2x magnification of the lumenal section of the intestine; n/a = not applicable. (TIF) [file pone.0129381.s004.tif]

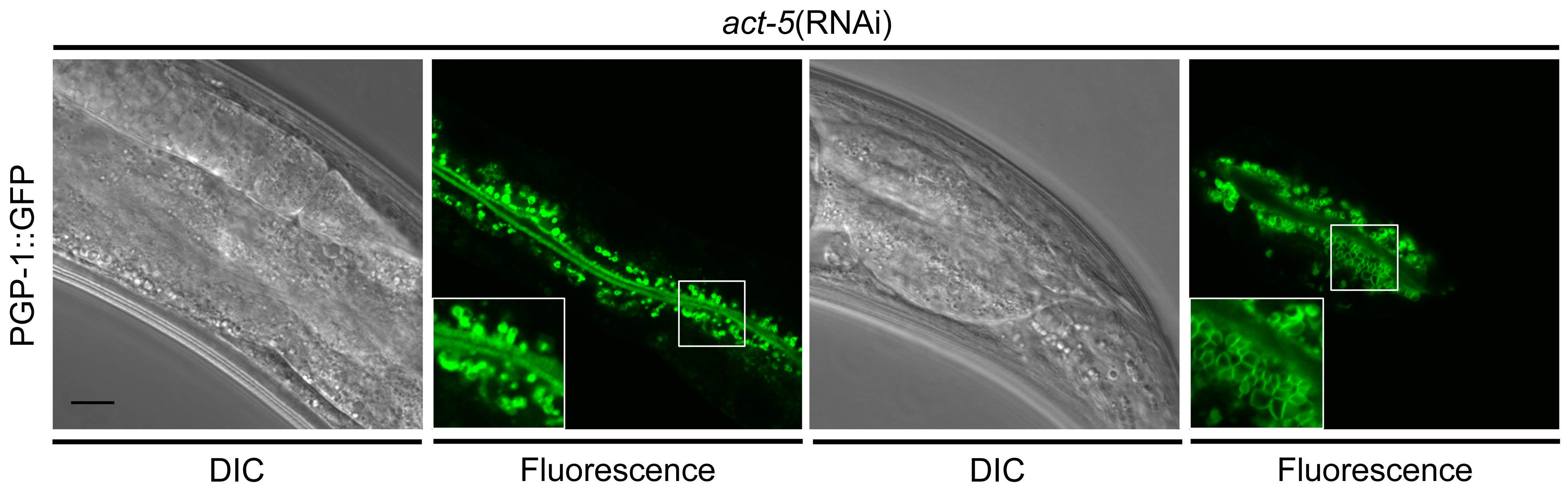

Supplement: S5 Fig — C. elegans L4 larvae expressing PGP-1::GFP were fed for 24 h with E. coli expressing act-5 dsRNA. Reduced ACT-5 abundance gave rise to a disturbed intestinal apical plasma membrane, in which the lining was still visible but “bubbles” were formed towards the cytoplasm. Two representative images are shown. Scale bar: 10 μm; inset: 2x magnification of the lumenal section of the intestine. (TIF) [file pone.0129381.s005.tif]
