# Supplementary material for: Disruption of the C. elegans Intestinal Brush Border by the Fungal Lectin CCL2 Phenocopies Dietary Lectin Toxicity in Mammals
Source: PLoS One. 2015 Jun 9;10(6):e0129381. doi: 10.1371/journal.pone.0129381 (PMC4461262; doi:10.1371/journal.pone.0129381)
Supplement: S2 Table — (DOCX) [file pone.0129381.s007.docx]

|  | **Relevant genotype** | **Source or reference** |
| --- | --- | --- |
| **Bacterial strains** |  |  |
| *E. coli* OP50 | Ura^-^ | [61] |
| *E. coli* BL21(DE3) | *F^-^ ompT hsdS_B_(r_B_^-^ m_B_^-^) gal dcm* (DE3) | Novagen Merck KGaA |
| *E. coli* JM109(DE3) | F’ (traD36, *pro*AB+ *lac*I^q^, Δ(*lac*Z)M15) *end*A1 *rec*A1 *hsd*R17(r_k_^-^, m_k_^+^) *mcr*A *sup*E44 λ^-^ *gyr*A96 *rel*A1 Δ(*lac-proAB*) *thi*-1, (DE3) | H. Schulenburg^1^ |
| *E. coli* HT115(DE3) | W3110, rnc14 :: ΔTn10 | [60] |
| *B. subtilis* 168 | *trpC2* | U. Sauer^2^ |
|  |  |  |
| ***C. elegans* strains** |  |  |
| N2 (Bristol type) | Wild type | CGC^3^ |
| GK70 | *dkIs37[P_act-5_::gfp::pgp-1]* | K. Sato [26] |
| GK173 | *dkIs92[P_vha-6_::gfp::rab-8]* | K. Sato [26] |
| VJ402 | *fgEx13[P_erm-1_::erm-1::gfp rol-6(su1006)]* | V. Göbel [28] |
| VJ268 | *fgEx12[P_act-5_::act-5::gfp]* | V. Göbel [29] |
| BJ49 | *kcIs6[P_ifb-2_::ifb-2::cfp]* | O. Bossinger [30] |
| WS649 | *opEx1532[P_ges-1_::bre-1::mCherry::ges-1(3’)]* | This study |
| WS6259 | *opEx1481[P_ges-1_::ger-1::mCherry::ges-1(3’)]* | This study |
| WS6227 | *opEx1555[P_ges-1_::fut-1::mCherry::ges-1(3’)]* | This study |
| WS6045^4^ | *bre-1(ye4)* | CGC^3^ |
| WS5864 | *ger-1(op499)* | A. Butschi [65] |
| VC585 | *fut-1(ok892)* | CGC^3^ |

^1^ Hinrich Schulenburg, Evolutionary Ecology Genetics, Zoological Institute CAU, Kiel D.

^2^ Uwe Sauer, Institute of Molecular Systems Biology, ETH Zurich, Zurich CH.

^3^ Caenorhabditis Genetics Center (CGC), University of Minnesota, Minneapolis USA.

^4^ HY496 (2x bc) from CGC backcrossed (bc) 2x to N2 to get WS6045 (4x bc).
