# Supplementary material for: Disruption of the C. elegans Intestinal Brush Border by the Fungal Lectin CCL2 Phenocopies Dietary Lectin Toxicity in Mammals
Source: PLoS One. 2015 Jun 9;10(6):e0129381. doi: 10.1371/journal.pone.0129381 (PMC4461262; doi:10.1371/journal.pone.0129381)
Supplement: S1 Text — Detailed protocol. (DOCX) [file pone.0129381.s008.docx]

For transmission electron microscopy (TEM) and focused-ion beam scanning electron microscopy (FIB-SEM), worms were frozen as follows: One *C. elegans* worm was suspended in a droplet of M9 buffer and then drawn into a cellulose capillary tube, which was immediately transferred to 1-hexadecene, cut to a piece of about 4 mm in length, and transferred into the 150 µm cavity of a 6 mm aluminum specimen carrier. Excess 1-hexadecene was sucked off with a filter paper and the empty space filled with 20% dextran (MW 39,000; Sigma-Aldrich, St. Louis, USA) in PBS (phosphate-buffered saline). Sandwich was completed with a flat 6 mm aluminum specimen carrier and frozen in a HPM 100 high-pressure freezing machine (Leica Microsystems, Vienna, Austria) with ethanol as synchronization fluid. Freeze-substitution was carried out in waterfree acetone with 1% OsO_4_ for 8 h at -90°C, 7 h at -60°C, 5 h at -30°C, 1 h at 0°C, with transition gradients of 30°C per h, followed by 30 min incubation at room temperature. Samples were rinsed twice with waterfree acetone, blockstained with 1% uranyl acetate in acetone (stock solution: 20% in methanol) for 1 h at 4°C, rinsed twice with waterfree acetone and embedded in Epon/Araldite (Sigma-Aldrich) (66% Epon/Araldite in acetone overnight at 4°C, 100% Epon/Araldite for 1 h at room temperature and polymerized at 60°C for 20 h). Thin sections were post-stained with Reynolds lead citrate and analyzed at 80 kV acceleration voltage using a CM100 transmission electron microscope (http://www.fei.com; Eindhoven, NL) equipped with a side mounted digital camera Orius 1000 (Gatan, Munich, Germany).

For FIB-SEM, a trimmed Epon/Araldite block containing a single worm was mounted on a regular SEM stub using conductive carbon and coated with 10 nm of carbon by electron beam evaporation to render the sample conductive. Ion milling and image acquisition was performed simultaneously in an Auriga 40 Crossbeam system (Zeiss, Oberkochen, Germany) using the FIBICS Nanopatterning engine (Fibics Inc., Ottawa, Canada). A large trench was milled at a current of 20 nA and 30 kV, followed by fine milling at 600 pA and 30 kV during image acquisition with an advance of 5 nm per image. Prior to starting the fine milling and imaging, a protective Platinum layer of approximately 300 nm was applied on top of the surface of the area of interest using the single gas injection system at the FIB-SEM. SEM images were acquired at 1.7 kV (60 µm aperture and high-current mode) using an in-lens energy selective backscattered electron detector (ESB) with a grid voltage of 1.4 kV, and a dwell time of 35 or 40 μs. The pixel size was set to 5 nm and tilt-corrected to obtain isotropic voxels. The final image stack was cropped to the area of interest using the ImageJ image-processing package [56]. Alignment of the image stack was performed with the Sift plugin [55]. Segmentation of microvilli was done with Imaris (Bitplane AG, Zurich, Switzerland) using the surpass surface tool with a surface grain size of 8 / 10 nm (control / CCL2) and manual thresholds for lower and upper gray levels of 103 / 140 and 132 / 214 (control / CCL2), respectively.
